# Shared and Distinct Fractional Amplitude of Low-Frequency Fluctuation Patterns in Major Depressive Disorders With and Without Gastrointestinal Symptoms

**DOI:** 10.3389/fpsyt.2021.744898

**Published:** 2021-12-02

**Authors:** Xiaoya Fu, Huabing Li, Meiqi Yan, Jindong Chen, Feng Liu, Jingping Zhao, Wenbin Guo

**Affiliations:** ^1^National Clinical Research Center for Mental Disorders, Department of Psychiatry, The Second Xiangya Hospital of Central South University, Changsha, China; ^2^Department of Radiology, The Second Xiangya Hospital of Central South University, Changsha, China; ^3^Department of Radiology, Tianjin Medical University General Hospital, Tianjin, China; ^4^Department of Psychiatry, The Third People's Hospital of Foshan, Foshan, China

**Keywords:** fractional amplitude low-frequency fluctuation, major depressive disorder, gastrointestinal symptoms, functional magnet resonance imaging (fMRI), somatic symptoms

## Abstract

**Objective:** Gastrointestinal (GI) symptoms are fairly common somatic symptoms in depressed patients. The purpose of this study was to explore the influence of concomitant GI symptoms on the fractional amplitude of low-frequency fluctuation (fALFF) patterns in patients with major depressive disorder (MDD) and investigate the connection between aberrant fALFF and clinical characteristics.

**Methods:** This study included 35 MDD patients with GI symptoms (GI-MDD patients), 17 MDD patients without GI symptoms (nGI-MDD patients), and 28 healthy controls (HCs). The fALFF method was used to analyze the resting-state functional magnetic resonance imaging data. Correlation analysis and pattern classification were employed to investigate the relationship of the fALFF patterns with the clinical characteristics of patients.

**Results:** GI-MDD patients exhibited higher scores in the HRSD-17 and suffered more severe insomnia, anxiety/somatization, and weight loss than nGI-MDD patients. GI-MDD patients showed higher fALFF in the right superior frontal gyrus (SFG)/middle frontal gyrus (MFG) and lower fALFF in the left superior medial prefrontal cortex (MPFC) compared with nGI-MDD patients. A combination of the fALFF values of these two clusters could be applied to discriminate GI-MDD patients from nGI-MDD patients, with accuracy, sensitivity, and specificity of 86.54, 94.29, and 70.59%, respectively.

**Conclusion:** GI-MDD patients showed more severe depressive symptoms. Increased fALFF in the right SFG/MFG and decreased fALFF in the left superior MPFC might be distinctive neurobiological features of MDD patients with GI symptoms.

## Introduction

As one of the most widespread mental illnesses across the world, major depressive disorder (MDD) is a serious public health problem and markedly impairs patient's quality of life. It is extremely common for patients with MDD to report somatic symptoms, such as constipation and stomachache. A multicenter study showed that half of MDD patients had multiple unexplained somatic symptoms (1). In elderly patients with MDD, the prevalence of somatic symptoms is even higher (2). Among these somatic symptoms, gastrointestinal (GI) symptom is prevalent in depressed patients and has a close connection to depression (3, 4). Over 70% of patients with depressive episodes reported concomitant GI symptoms (5). Patients with GI symptoms also have a greater probability of suffering from severe depression or anxiety (5, 6).

Early and accurate diagnosis of MDD is a matter of great importance to optimize patient outcomes. However, the existence of somatic symptoms in MDD patients makes it more difficult to recognize the psychological symptoms. According to the results of an international study across 15 centers in 14 countries, about 69% of patients with MDD only sought medical care for somatic symptoms, and 11% of patients denied depressed mood and feelings of guilt or worthlessness, which are the two most significant symptoms to the diagnosis of MDD (1). GI symptoms are common in primary care, but often, no pathological cause can be found. Functional gastrointestinal disorders (FGIDs) refer to chronic or recurrent GI symptoms without identified structural or biochemical abnormalities, such as irritable bowel syndrome (IBS) and functional dyspepsia (7). It is widely acknowledged that FGIDs are closely related to depression (8). Tricyclic antidepressants and serotonin noradrenergic reuptake inhibitors are recommended in the treatment of chronic GI pain and painful FGIDs (9). GI comorbidities might result in delays in accurate diagnosis and effective treatment for MDD. Moreover, patients with MDD often bounce from one specialist to another in search of a diagnosis of physical diseases because of the co-occurrence of GI symptoms, which also imposes great economic hardship on patients. A review reported that 6–38.5% of clinic patients with IBS have a diagnosis of depressive disorders (10). About 29% of patients with unexplained GI symptoms referred to upper endoscopy were detected as patients with MDD (11).

The close association between GI symptoms and depression indicates a connection between the pathological mechanisms of GI symptoms and depression. Numerous studies have suggested the significance of the gut–brain axis on human psychiatric health (12). The gut–brain axis, which refers to the bidirectional information transfer between the GI tract and the nervous system, is of great importance to normal healthy homeostasis (13). The gut microbial dysbiosis can participate in mental disorders through numerous pathways, such as the autonomic nerve system, neuroendocrine system, and the immune system (14). Therefore, GI symptoms might be the manifestations of gut microbiota dysfunction, which could show associations with functional changes in the brain. Studies on FGIDs exhibited evidence supporting this idea that functional GI symptoms are related to functional changes in the brain. Patients with functional dyspepsia manifested altered functional connectivity (FC) in the amygdala and insula (15, 16). Reduced FC between the hypothalamus and high-order cortical area was found in adolescent IBS patients (17). Compared to female healthy controls, female IBS patients showed altered FC of the dorsal anterior insular with the medial prefrontal cortex (MPFC) and precuneus (18). Nevertheless, only a few previous studies have focused on functional alterations related to comorbid GI symptoms in MDD patients (19, 20). Studies using regional homogeneity (ReHo) have found functional alterations of MDD patients with concomitant GI symptoms chiefly in the frontal lobe, precentral gyrus, and superior temporal gyrus, as well as the precuneus relative to MDD patients without GI symptoms (19, 20).

Investigating the brain functional changes of depressed patients with GI symptoms is beneficial in order to obtain an understanding of the pathophysiology behind it. While ReHo reflects the regional property of spontaneous neural activity to some degree, it is based on the temporal similarity of spontaneous brain activity of spatially adjacent voxels. Knowledge of the amplitude of regional neural activity in MDD patients with GI symptoms is still limited. The amplitude of low-frequency fluctuation (ALFF), correlated with cerebral blood flow, is considered to reflect the strength of the low-frequency fluctuation of spontaneous neural activity (8, 9). To diminish the effect of physiological noise in ALFF analysis, Zou et al. (21) proposed fractional ALFF (fALFF) (10). ReHo and fALFF explore different aspects of spontaneous brain activity. Although similar trends of ReHo and fALFF have been shown in previous studies, opposite trends have also been reported (7). Therefore, it is significant to evaluate the effect of GI comorbidity on MDD from another aspect of spontaneous brain activity.

In this study, we explored the effect of concomitant GI symptoms on the clinical manifestations of MDD. Moreover, fALFF was employed to explore the shared and distinct patterns of functional changes in MDD patients with and without GI symptoms.

## Methods

### Participants

This study involved 52 first-episode, treatment-naive patients with MDD and 28 age-, gender-, and education-matched healthy controls (HCs). MDD patients were divided into GI-MDD patients (patients with GI symptoms, *n* = 35) and nGI-MDD patients (patients without GI symptoms, *n* = 17) based on whether they had GI symptoms or not. These GI symptoms mainly incorporate medically unexplained nausea, vomiting, heartburn, flatulence, gastralgia, constipation, diarrhea, etc. The participants were all Han Chinese and right-handed. Detailed demographic characteristics of the three groups are presented in Table 1. The diagnosis of MDD was made by two psychiatrists independently according to the Diagnostic and Statistical Manual of Mental Disorders, 5th edition (DSM-5). All patients had no psychotic symptoms and obtained a total score in the 17-item Hamilton Rating Scale for Depression (HRSD-17) of ≥17. Patients had no history of antidepressant use or electroconvulsive therapy. HCs never had psychotic symptoms or a history or family history of psychiatric disorders. For all participants, the exclusion criteria included other psychiatric disorders following the DSM-5 diagnostic criteria, digestive diseases with structural or organic pathology, a history of substance abuse/neurological conditions/severe physical diseases, brain structural abnormalities, pregnancy, or inability to participate in brain MRI scan.

**Table 1:** Demographic and clinical characteristics of the participants.

	**GI-MDD (*n* = 35)**	**nGI-MDD (*n* = 17)**	**HCs (*n* = 28)**	***F, t*, or χ^**2**^ value**	***post-hoc t*-test or *p*-value**
Age (years)	30.86 ± 6.84	30.29 ± 8.05	30.14 ± 5.00	0.102	0.903^a^
Sex (male/female)	13/22	6/11	14/14	1.377	0.502^b^
Handedness (right/left)	35/0	17/0	28/0		
Education (years)	14.51 ± 3.28	12.94 ± 3.46	14.61 ± 2.69	1.797	0.173^a^
Illness duration (months)	6.23 ± 4.63	6.94 ± 3.98		0.544	0.589^c^
HRSD-17 scores	22.69 ± 3.41	20.18 ± 2.67	0.89 ± 0.88	585.979	GI-MDD > nGI-MDD > HCs
Retardation symptoms	6.40 ± 1.42	6.76 ± 1.56	0.18 ± 0.39	253.030	GI-MDD, nGI-MDD > HCs
Cognitive disturbances	3.71 ± 1.78	3.41 ± 1.50	0	64.213	GI-MDD, nGI-MDD > HCs
Insomnia	4.46 ± 1.42	3.53 ± 1.28	0.32 ± 0.55	103.570	GI-MDD > nGI-MDD > HCs
Anxiety/somatization	7.31 ± 1.92	6.41 ± 1.66	0.39 ± 0.57	174.531	GI-MDD > nGI-MDD > HCs
Weight loss	0.80 ± 0.83	0.06 ± 0.24	0	18.741	GI-MDD > nGI-MDD, HCs

*HRSD-17, 17-item Hamilton Rating Scale for Depression; GI-MDD, MDD with gastrointestinal (GI) symptoms; nGI-MDD, MDD without GI symptoms; HCs, healthy controls*.

a *The p-value was obtained using analysis of variance*.

b *The p-value was obtained using a chi-square test*.

c *The p-value was obtained using a two-sample t-test*.

The severity of the GI symptoms was evaluated by the GI symptoms item in HRSD-17. The severity of the clinical symptoms of MDD patients was assessed by the scores of HRSD-17 and the following aspects in HRSD-17: retardation symptoms (items 1, 7, 8, and 14), cognitive disturbances (items 2, 3, and 9), insomnia (items 4, 5, and 6), anxiety/somatization (items 10–13, 15, and 17), and weight loss (item 16).

All participants provided written informed consent. This study conformed to the standards of the Declaration of Helsinki and was approved by the Medical Research Ethics Committee of the Second Xiangya Hospital of Central South University.

### Image Acquisition

All participants underwent scanning on a Siemens 3.0-T scanner. Participants were requested to close their eyes and stay awake during the scan. Headphones and foam padding were used to restrict head motion and minimize scanner noise. Echo planar imaging (EPI) sequence was employed when obtaining resting-state functional magnetic resonance images (fMRI) data [repetition time (TR)/echo time (TE) = 2,000 ms/30 ms, flip angle = 90°, field of view = 240 × 240 mm, matrix = 64 × 64, 4-mm slice thickness, 0.4-mm gap, 30 slices, number of volumes = 250].

### Imaging Data Processing

Data preprocessing was performed using the Data Processing Assistant for Resting-State fMRI (DPARSF v5.2; http://rfmri.org/DPARSF) software package (22). The initial 10 volumes of each participant were deleted for the MRI signal to reach a steady state and for the participants to adapt to the scanning noise. After slice timing correction, head motion correction was conducted; participants with head motion exceeding 2 mm of the maximum displacement or 2° of the maximal rotation were excluded. Subsequently, these images were spatially normalized to the Montreal Neurological Institute (MNI) space and resampled to a resolution of 3 × 3 × 3 mm^3^. Spatial smoothing was conducted with a 4-mm Gaussian kernel of full width at half maximum (FWHM). The calculation of fALFF has been published in previous research (21). Fast Fourier transform was used to convert the time series into the frequency domain to generate the power spectrum. Then, the square root of the power spectrum was calculated and then averaged across the frequency of 0.01–0.08 Hz. The fALFF was the ratio of the sum of amplitude across 0.01–0.08 Hz to that across the entire frequency range. The fALFF of each voxel was divided by the global mean fALFF for standardization.

### Statistical Analysis

We compared the demographic, clinical, and neuroimaging data across GI-MDD patients, nGI-MDD patients, and HCs. Student's *t*-tests or one-way analyses of variance (ANOVA) was used to compare continuous data. Categorical data were analyzed with a chi-square test. Analysis of covariance (ANCOVA) was adopted to analyze fALFF, and age, gender, years of education, and the mean framewise displacement (FD) were set as covariates. *Post*-*hoc t*-test was performed for multiple comparisons. Differences were considered significant at a false discovery rate (FDR)-corrected *p* < 0.05. The fALFF values of clusters with significant differences between groups were extracted for further correlation and classification analysis.

After assessing the normal distribution of the data, we analyzed the correlation between the extracted fALFF and the scores of HRSD-17 and its subscales. Benjamini–Hochberg correction was used in the correlation analysis.

To test the capability of extracted fALFF to discriminate between GI-MDD patients and nGI-MDD patients, the support vector machine (SVM) method was applied. SVM is a common supervised machine learning model that can be applied to explore the best boundary between two categories to solve a binary classification problem. The analysis used a “leave-one-out” method and was conducted using the LIBSVM software package (23) in MATLAB.

## Results

### Demographic and Clinical Data

No participant was excluded because of excessive head motion. Table 1 presents the demographic and clinical data of the three groups. There was no significant difference in age, gender, or years of education across the three groups. GI-MDD patients and nGI-MDD patients did not differ in the duration of illness. GI-MDD patients had higher HRSD-17 total scores and scored higher with respect to insomnia, anxiety/somatization, and weight loss than did nGI-MDD patients. Except for the weight loss subscale, both patient groups exhibited higher scores in the HRSD-17 scale and four other subscales relative to HCs.

### Group Differences in fALFF

**Figure 1:**
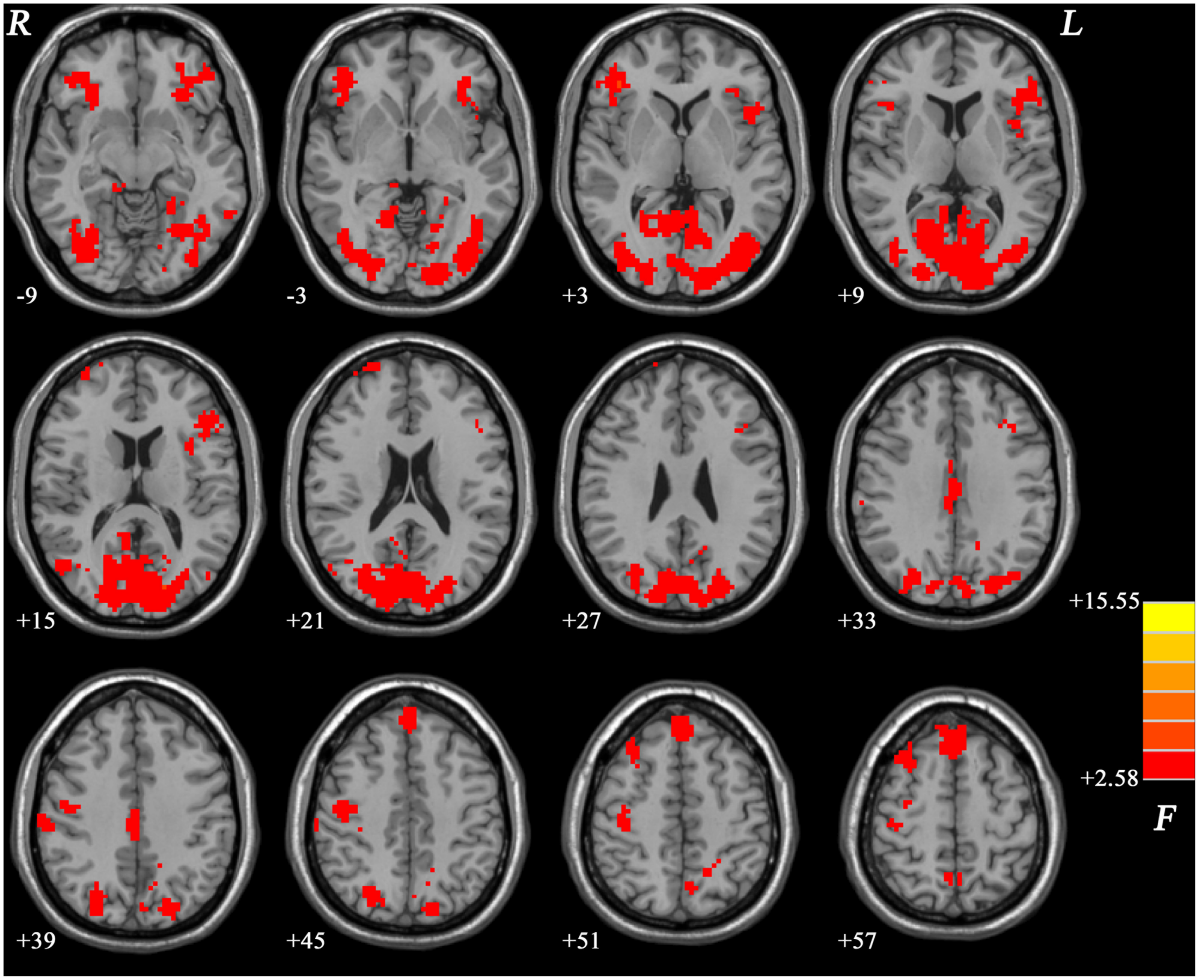
Brain regions showing significantly different fALFF values across three groups at a false discovery rate (FDR)-corrected *p* < 0.05. The *color bar* indicates the *F* values based on ANCOVA. *fALFF*, fractional amplitude of low-frequency fluctuation; *ANCOVA*, analysis of covariance.

We compared individual whole-brain fALFF across GI-MDD patients, nGI-MDD patients, and HCs. Significant differences in fALFF were displayed mainly in the frontal and occipital regions (Figure 1).

#### fALFF Differences Between GI-MDD Patients and nGI-MDD Patients

**Figure 2:**
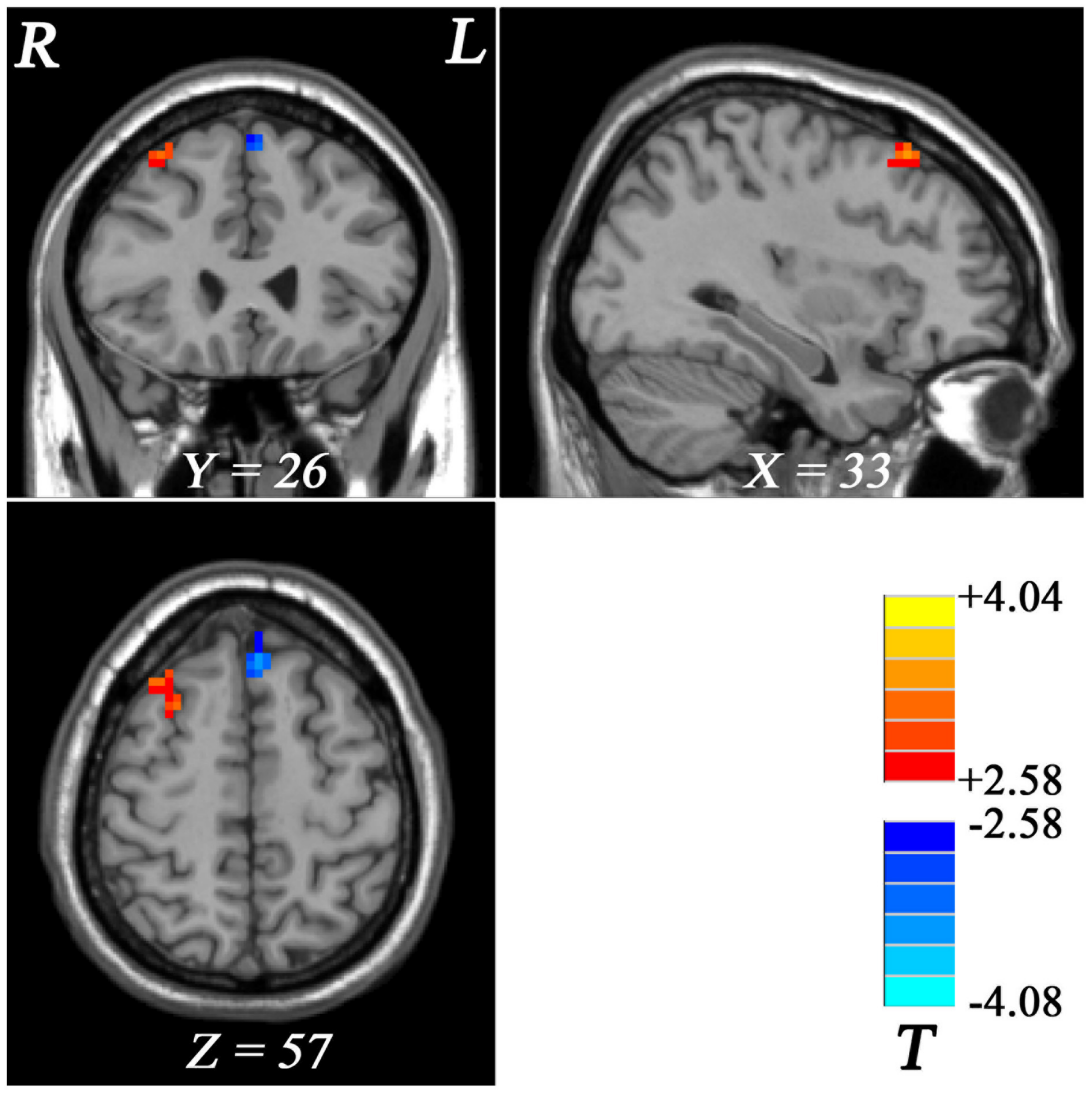
Regional fALFF differences between GI-MDD patients and nGI-MDD patients. The *color bar* indicates the *t* values from *post-hoc t*-tests. *Red* and *blue colors* denote increased and decreased fALFF, respectively. *fALFF*, fractional amplitude of low-frequency fluctuation; *GI-MDD*, major depressive disorder with gastrointestinal symptoms; *nGI-MDD*, major depressive disorder without gastrointestinal symptoms.

Compared with nGI-MDD patients, GI-MDD patients exhibited increased fALFF in the right superior frontal gyrus (SFG)/middle frontal gyrus (MFG) and decreased fALFF in the left superior MPFC (Figure 2, Table 2). There was no other significant difference in the fALFF between the two patient groups. Considering the potential effect that the severity of depression might be a confounding factor in the comparison, we further added the total score of HRSD-17 as a covariate in the between-group comparison and obtained similar results, suggesting that the severity of depression had limited effects on the results (Figure S1, Table S1).

**Table 2:** Significant fractional amplitude of low-frequency fluctuation (fALFF) differences across three groups.

**Cluster location**	**Peak (MNI)**			**No. of voxels**	***t* value**
	**x**	**y**	**z**		
**GI-MDD vs. nGI-MDD**					
Right superior frontal gyrus/middle frontal gyrus	33	24	54	35	3.3962
Left superior MPFC	−3	30	57	28	−3.5590
**GI-MDD vs. HCs**					
Right middle frontal gyrus/inferior frontal gyrus	39	36	0	55	5.1164
Right fusiform	36	−63	−15	47	−4.4455
Left cuneus	−18	−84	15	132	−4.6258
**nGI-MDD vs. HCs**					
Bilateral superior MPFC	0	39	57	178	5.1691
Right middle occipital gyrus/inferior occipital gyrus	36	−81	−9	50	−4.0813
Left middle occipital gyrus/inferior occipital gyrus	−36	−84	−3	106	−4.5530
Bilateral cuneus	0	−87	21	157	−4.6956

*MNI, Montreal Neurological Institute; MPFC, medial prefrontal cortex; GI-MDD, MDD with gastrointestinal (GI) symptoms; nGI-MDD, MDD without GI symptoms; HCs, healthy controls*.

#### fALFF Differences Between GI-MDD Patients and HCs

**Figure 3:**
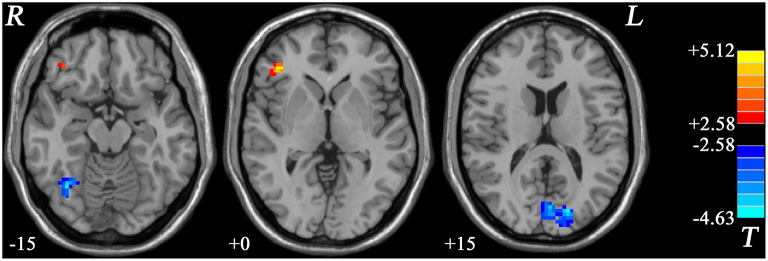
Statistical maps showing the fALFF differences between GI-MDD patients and HCs. The *color bar* indicates *t* values from *post-hoc t*-tests. *Red* and *blue colors* denote increased and decreased fALFF, respectively. *fALFF*, fractional amplitude of low-frequency fluctuation; *GI-MDD*, major depressive disorder with gastrointestinal symptoms; *HCs*, healthy controls.

In GI-MDD patients, increased fALFF was found in the right MFG/inferior frontal gyrus (IFG) compared to HCs. Also, decreased fALFF was present in the right fusiform and left cuneus in GI-MDD patients (Figure 3, Table 2).

#### fALFF Differences Between nGI-MDD Patients and HCs

**Figure 4:**
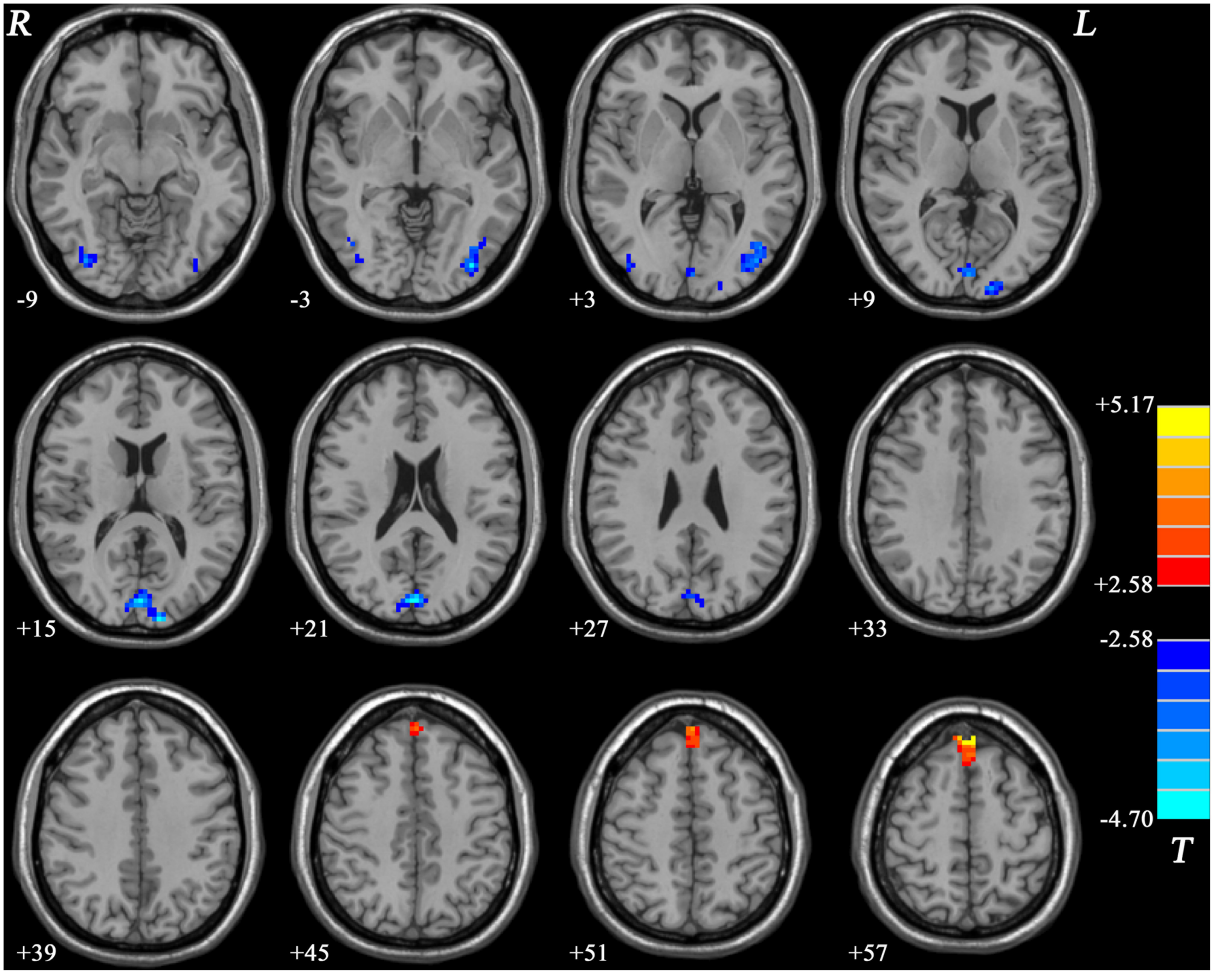
Statistical maps showing the fALFF differences between nGI-MDD patients and HCs. The *color bar* indicates *t* values from *post-hoc t*-tests. *Red* and *blue colors* denote increased and decreased fALFF, respectively. *fALFF*, fractional amplitude of low-frequency fluctuation; *nGI-MDD*, major depressive disorder without gastrointestinal symptoms; *HCs*, healthy controls.

Compared with HCs, nGI-MDD patients showed higher fALFF values in the bilateral superior MPFC. Moreover, decreased fALFF was shown in nGI-MDD patients in the bilateral middle occipital gyrus (MOG)/inferior occipital gyrus (IOG), as well as in the bilateral cuneus (Figure 4, Table 2).

### Correlation Analysis

After assessment of normality, correlation analysis was conducted between the fALFF values and the scores of the HRSD-17, the scores of five subscales, and the severity of GI symptoms.

**Figure 5:**
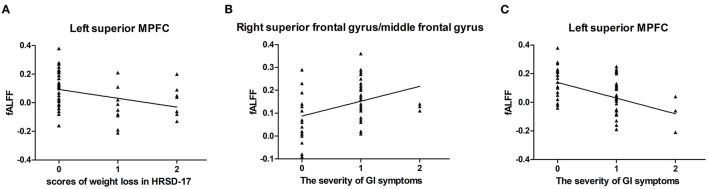
Correlations between abnormal fALFF and clinical variables. For all MDD patients, a negative correlation was found between the scores of weight loss in HRSD-17 and the fALFF of the left superior MPFC **(A)**. The severity of GI symptoms was found positively correlated with the fALFF values of the right superior frontal gyrus/middle frontal gyrus **(B)** and negatively correlated with the fALFF values of the left superior MPFC **(C)**. *fALFF*, fractional amplitude of low-frequency fluctuation; *MDD*, major depressive disorder; *HRSD-17*, 17-item Hamilton Rating Scale for Depression; *MPFC*, medial prefrontal cortex.

For all MDD patients, the fALFF values of the left superior MPFC showed an inverse correlation with the score of weight loss (*r* = −0.404, *p* = 0.003, corrected *p* = 0.021) (Figure 5A). In addition, the results showed that the severity of GI symptoms were positively correlated with fALFF in the right SFG/MFG (*r* = 0.380, *p* = 0.005, corrected *p* = 0.023) (Figure 5B) and negatively correlated with fALFF in the left superior MPFC (*r* = −0.438, *p* = 0.001, corrected *p* = 0.014) (Figure 5C).

For GI-MDD patients, the fALFF in the left superior MPFC was positively correlated with the total scores of HRSD-17 (*r* = 0.356, *p* = 0.036) and the scores in the anxiety/somatization aspect (*r* = 0.377, *p* = 0.025), but these correlations did not survive after correction.

No correlation was discerned in nGI-MDD patients between the fALFF values and the scores of the HRSD-17 or its subscales.

### SVM Results

**Figure 6:**
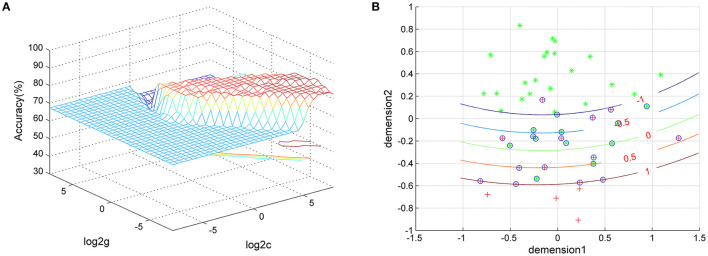
Visualization of the classification using the combination of fALFF values of the left superior MPFC and the right superior frontal gyrus/middle frontal gyrus through SVM. **(A)** SVM parameter results with 3D view. **(B)** Classified map of the results. *Green crosses* represent GI-MDD patients and *red crosses* represent GI-MDD patients. *fALFF*, fractional amplitude of low-frequency fluctuation; *MPFC*, medial prefrontal cortex; *SVM*, support vector machine; *GI-MDD*, major depressive disorder with gastrointestinal symptoms; *nGI-MDD*, major depressive disorder without gastrointestinal symptoms.

We used SVM classifiers to explore features that could distinguish between GI-MDD patients and nGI-MDD patients. The clusters exhibiting significantly different fALFF values (right SFG/MFG and left superior MPFC), separately or together, were used as features. The results showed that classification based on the combination of the fALFF values in the right SFG/MFG and the left superior MPFC reached a higher accuracy (86.54%) than did those based on fALFF of the right SFG/MFG (76.92%) or the left superior MPFC (84.62%) alone. When using the fALFF values of the right SFG/MFG as the feature, the sensitivity and specificity were 94.29 and 41.18%, respectively. The sensitivity and specificity were 100 and 58.82%, respectively, when using the fALFF values of the left superior MPFC to discriminate between GI-MDD patients and nGI-MDD patients. The combination of the fALFF values of these two regions exhibited sensitivity and specificity of 94.29 and 70.59%, respectively (Figure 6).

## Discussion

This research showed that GI-MDD patients suffered a higher level of depression than nGI-MDD patients, especially in terms of insomnia, anxiety/somatization, and weight loss, which reinforced the negative effect of concomitant GI symptoms on MDD patients. More importantly, the fMRI results revealed that GI-MDD patients exhibited increased fALFF in the right SFG/MFG and decreased fALFF in the left superior MPFC compared to nGI-MDD patients. The SVM analysis exhibited that a combination of the fALFF values of these two regions could discriminate between GI-MDD patients and nGI-MDD patients with accuracy, sensitivity, and specificity of 86.54, 94.29, and 70.59%, respectively.

Our results found that GI-MDD patients obtained higher scores in the HRSD-17 and its subscales on insomnia, anxiety/somatization, and weight loss, which suggested that GI symptoms were related to a higher level of depression. Other research papers have reported similar results (5, 6, 24). A multicenter study found that GI symptoms made patients almost five times more likely to be subject to severe depression and nearly four times more likely to undergo severe anxiety (6). Another research studying 3,256 MDD patients in China reported that increased frequency of GI symptoms showed a correlation with higher possibilities of anxiety, depression, and insomnia (5). In patients with FGIDs, as the number of FGIDs and the severity and frequency of the GI symptoms increased, the risk of depression and anxiety increased in a stepwise manner (24). These studies indicated that concomitant GI symptoms would negatively influence the course of MDD. The gut–brain axis concept provides a possible Mechanism by which gut microbiota could play a crucial role in the development of diseases, including depression. The two-way communication between the GI tract and the brain has been widely recognized. For instance, the gut microbiota can regulate the function of the hypothalamic–pituitary–adrenal (HPA) axis and directly influence the function of the central neural system through the activation of neurons in the stress circuits (12). Some experiments and clinical trials have found that probiotic treatment is helpful in reducing depressive behavior (25–27). Therefore, the management of GI symptoms is of great clinical significance in the treatment and prognosis of MDD.

The fALFF, related to cerebral blood flow, reflects the spontaneous neuronal activity. In this study, GI-MDD patients displayed increased fALFF in the right SFG/MFG compared with nGI-MDD patients. For all MDD patients, the fALFF values of the right SFG/MFG positively correlated with the severity of GI symptoms. The results also showed that fALFF was higher in the right MFG/IFG in GI-MDD patients compared to HCs. These findings suggested that the frontal lobe, especially the MFG, might have a close connection to GI symptoms in MDD. The study of Geng et al. showed altered ReHo in the right MFG in MDD patients with somatic symptoms (28). In GI-MDD patients, decreased gray matter volume (GMV) and ReHo in the right SFG and MFG were also found (19). It is considered that the MFG is critical for bottom-up sensory-driven exogenous attention. Reorienting to unpredicted stimuli can activate the right MFG (29). Some researchers have proposed that the MFG serves as the gatekeeper between the dorsal attention network and the ventral attention network, and it could interact with both networks and interrupt processes of goal-directed attention to reorient to stimulus-driven attention (30). It is speculated that patients with FGIDs selectively attend to GI sensations, and this selective attention or hypervigilance may lead to hyperalgesia (31). Patients with IBS relative to HCs exhibited greater brain response in the MFG when they were in contextual threat of abdominal electrical stimulation, suggesting inappropriate allocation of attentional resources (32). Therefore, abnormal fALFF in the right MFG might be associated with hypervigilance and attentional bias toward visceral interoceptive sensations.

Numerous studies have supported the connection between depression and the default-mode network (DMN), which includes the MPFC, posterior cingulate cortex, and the inferior parietal lobe (33, 34). Among these regions, MPFC is closely related to chronic stress and self-focus, both of which are risk factors of depression (35–37). We found decreased fALFF in the left superior MPFC in GI-MDD patients compared to nGI-MDD patients. The fALFF values of the left superior MPFC showed inverse correlations with the scores of weight loss and the severity of GI symptoms. Also, a higher fALFF was exhibited in the bilateral superior MPFC in nGI-MDD patients compared to HCs. These results implied that MPFC was not only related to MDD but also involved in the GI symptoms of MDD. MPFC has gained consensus for having an involvement in MDD (38). Our previous research reported that the left superior MPFC showed an increased DMN homogeneity in first-episode, drug-naive MDD patients, and this finding was replicated in two other separate but similar samples (39, 40). Thus, an altered functional activity in the left superior MPFC might reflect the core neuropathological mechanism in MDD. It is widely considered that self-focus features prominently in patients with MDD, accompanied by increased rumination thinking and negative affectivity (41, 42). MPFC is of great importance in processing emotion and self-related information (43, 44). A meta-analysis exploring the neural substrates of rumination found that rumination-related hyperactivation included the core and the dorsal MPFC subsystems of the DMN (45). Moreover, impaired function of the MPFC, together with the dorsolateral prefrontal cortices, was associated with deficiencies in executive function and effortful regulation of behavior and emotional state, which might lead to depressed mood and anhedonia (46). These findings indicated that abnormal functional activity of the left superior MPFC might be a possible biomarker for MDD.

The MPFC was reported to have portions of cortical neurons that control the parasympathetic output of the stomach (47). In addition, the MPFC was found to be related to the development of stress-induced gastric mucosal lesion (SGML) (48). This might be the reason for the abnormal functional activity of MPFC having been observed in mental illness with somatic symptoms, including GI symptoms. Increased connectivity between the left superior MPFC and the lobule IX was found in patients with somatization disorder (49). Patients with somatization disorder had increased fALFF in the bilateral superior MPFC compared to HCs (50). A study investigating the functional activity of the brain in hyperalgesia reported increased activity in the MPFC in central sensitization, which is a state of high reactivity of the nervous system resulting in hypersensitivity to pain, and this hyperactivity could be suppressed with antihyperalgesic treatment (51). It seems to be in line with our results as some studies have proposed that FGIDs are often comorbid with hyperalgesia (38). In a paper on the abnormalities of regional brain activity in IBS, decreased ALFF in the MPFC and altered functional connectivity of the MPFC were found in IBS patients, and the aberrant ALFF was not eliminated when anxiety and depression were set as covariates (52). Therefore, a decreased ALFF in the left superior MPFC might have a connection with hyperalgesia, which could be induced by chronic psychological stress and serve as an important pathophysiological component underlying FGIDs (53–56).

Decreased fALFF was shown in nGI-MDD patients in the bilateral cuneus and the bilateral MOG/IOG compared to HCs. Decreased fALFF has also been found in the left cuneus in GI-MDD patients compared to HCs, which suggested that the hypoactivity of these regions was important in the pathogenesis of MDD. Structural and functional changes of the occipital lobe are not rare in MDD patients. Reductions in the volume of the bilateral MOG and the right IOG have been described in MDD (57). A study interested in occipital bending, a type of structural asymmetry of the brain where one occipital lobe wraps across the midline, found that the prevalence of occipital bending was three times higher in MDD patients than in controls (58). As for the functional abnormality of the occipital lobes, it was reported that both unipolar depression and bipolar depression shared ReHo changes in the cuneus (59). Decreased fALFF in the occipital cortex was also found in our previous study on MDD patients (60). In addition, a graph-based analysis study revealed that MDD patients showed abnormal nodal degree in the occipital cortex, suggesting altered regional connectivity of the occipital cortex (61). It has been reported that the γ-aminobutyric acid (GABA) levels in the occipital cortex decreased in MDD patients and that selective serotonin reuptake inhibitors (SSRIs) could reverse this reduction (62, 63). Moreover, the occipital lobe contributes to visual-induced emotional information processing and the perception of facial emotion (44, 64). Depressive adolescents exhibited distorted processing of emotion- and self-related visual information (65). Chechko et al. found that the bilateral MOG and IOG showed greater activation in an emotional conflict task (66). However, unmedicated patients with MDD showed poor accuracy and lower functional activity in the MOG and IOG in an emotional conflict task (64). Therefore, our findings, in line with these studies, suggested that aberrant activity in the occipital cortex was possibly related to the disrupted visual-induced emotional information processing in MDD patients.

The right fusiform gyrus only exhibited decreased fALFF in GI-MDD patients compared to HCs. The fusiform gyrus is an important structure in processing high-order visual information such as face perception (67). The study of Liu et al. has reported increased ReHo in the right fusiform gyrus in GI-MDD patients (19). But the findings on the right fusiform gyrus in patients comorbid with MDD and GI symptoms were not consistent (20). A study on brain structural alterations reported that patients without GI symptoms displayed increased regional GMV and gray matter density (GMD) in the right fusiform gyrus compared to HCs (68). Thus, the association between GI symptoms in MDD and the fusiform is still vague. But several studies found structural or functional deficits of the fusiform gyrus in MDD patients (69–72). An imaging meta-analysis also reported decreased ALFF in the right fusiform gyrus in MDD patients (73).

We should note some limitations. Firstly, we did not further categorize patients according to their GI symptoms to discover the distinction of the brain functional changes between patients with different GI symptoms because of the small sample size. Secondly, only one item in the HRSD-17 was used to assess the severity of GI symptoms. Although some previous works have also used this evaluation approach (1, 2), it would be better to use a more specific scale to evaluate GI symptoms. Thirdly, this is a cross-sectional study, so it is a theme worth discussing that the abnormality of fALFF is the driver of GI symptoms in MDD or a consequence. Longitudinal research is needed to deepen the awareness of the pathophysiological features of MDD co-occurring with GI symptoms.

## Conclusion

This study reinforced the negative effect of concomitant GI symptoms on MDD patients. Our findings exhibited the shared and distinct patterns of functional changes in MDD patients with and without GI symptoms. The fALFF values in the right SFG/MFG and the left superior MPFC were distinct between MDD patients with and without GI symptoms, which suggested a possible association of the functional activity in these regions with MDD-related GI dysfunction.

## Data Availability Statement

The data in this study are available upon request to the corresponding author.

## Ethics Statement

The studies involving human participants were reviewed and approved by Medical Research Ethics Committee of the Second Xiangya Hospital of Central South University. The patients/participants provided their written informed consent to participate in this study.

## Author Contributions

XF wrote the manuscript. HL conducted the study. MY, JC, FL, and JZ contributed to managing and analyzing the imaging data. WG designed the study and analyzed the data. All authors contributed to and approved the final manuscript.

## Funding

This study was supported by grants from the National Key R&D Program of China (Grant No. 2016YFC1307100), the National Natural Science Foundation of China (Grant No. 81771447), the Natural Science Foundation of Hunan (Grant No. 2020JJ4784), Science and Technology Program of Hunan Province (Grant No. 2020SK53413), Key-Area Research and Development Program of Guangdong Province (2018B030334001), and the Natural Science Foundation of Tianjin (Grant No. 18JCQNJC10900).

## Conflict of Interest

The authors declare that the research was conducted in the absence of any commercial or financial relationships that could be construed as a potential conflict of interest.

## Publisher's Note

All claims expressed in this article are solely those of the authors and do not necessarily represent those of their affiliated organizations, or those of the publisher, the editors and the reviewers. Any product that may be evaluated in this article, or claim that may be made by its manufacturer, is not guaranteed or endorsed by the publisher.

## References

[1] Simon GE VonKorff M Piccinelli M Fullerton C Ormel J. An international study of the relation between somatic symptoms and depression. N Engl J Med. (1999) 341:1329–35. 10.1056/NEJM199910283411801 10536124

[2] Grover S Sahoo S Chakrabarti S Avasthi A. Anxiety and somatic symptoms among elderly patients with depression. Asian J Psychiatr. (2019) 41:66–72. 10.1016/j.ajp.2018.07.009 30054249

[3] Avramidou M Angst F Angst J Aeschlimann A Rössler W Schnyder U. Epidemiology of gastrointestinal symptoms in young and middle-aged Swiss adults: prevalences and comorbidities in a longitudinal population cohort over 28 years. BMC Gastroenterol. (2018) 18:21. 10.1186/s12876-018-0749-3 29374473 PMC5787318

[4] Huang M-H Wang Y-P Wu P-S Chan Y-LE Cheng C-M Yang C-H. Association between gastrointestinal symptoms and depression among older adults in Taiwan: a cross-sectional study. J Chin Med Assoc. (2021) 84:331–5. 10.1097/JCMA.0000000000000460 33186213 PMC12966144

[5] Huang J Cai Y Su Y Zhang M Shi Y Zhu N. gastrointestinal symptoms during depressive episodes in 3256 patients with major depressive disorders: findings from the NSSD. J Affect Disord. (2021) 286:27–32. 10.1016/j.jad.2021.02.039 33667753

[6] Mussell M Kroenke K Spitzer RL Williams JBW Herzog W Löwe B. Gastrointestinal symptoms in primary care: prevalence and association with depression and anxiety. J Psychosom Res. (2008) 64:605–12. 10.1016/j.jpsychores.2008.02.019 18501261

[7] Corazziari E. Definition and epidemiology of functional gastrointestinal disorders. Best PractRes Clin Gastroenterol. (2004) 18:613–31. 10.1016/j.bpg.2004.04.012 15324703

[8] Mayer EA Craske M Naliboff BD. Depression, anxiety, and the gastrointestinal system. J Clin Psychiatry. (2001) 8:28–36. 12108819

[9] Drossman DA Tack J Ford AC Szigethy E Törnblom H Van Oudenhove L. Neuromodulators for functional gastrointestinal disorders (disorders of gut–brain interaction): a rome foundation working team report. Gastroenterology. (2018) 154:1140–71. 10.1053/j.gastro.2017.11.279 29274869

[10] Creed F. The relationship between psychosocial parameters and outcome in irritable bowel syndrome. Am J Med. (1999) 107:74–80. 10.1016/S0002-9343(99)00083-2 10588176

[11] O'Malley PG Wong PWK Kroenke K Roy MJ Wong RKH. The value of screening for psychiatric disorders prior to upper endoscopy. J Psychosom Res. (1998) 44:279–87. 10.1016/S0022-3999(97)00250-X 9532557

[12] Foster JA McVey Neufeld K-A. Gut-brain axis: how the microbiome influences anxiety and depression. Trends Neurosci. (2013) 36:305–12. 10.1016/j.tins.2013.01.005 23384445

[13] Cryan JF O'Riordan KJ Cowan CSM Sandhu KV Bastiaanssen TFS Boehme M. The microbiota-gut-brain axis. Physiol Rev. (2019) 99:1877–2013. 10.1152/physrev.00018.2018 31460832

[14] Bruce-Keller A Salbaum JM Berthoud H-R. Harnessing gut microbes for mental health: getting from here to there. Biol Psychiatry. (2018) 83:214–23. 10.1016/j.biopsych.2017.08.014 29031410 PMC5859957

[15] Liu P Fan Y Wei Y Zeng F Li R Fei N. Altered structural and functional connectivity of the insula in functional dyspepsia. Neurogastroenterol Motil. (2018) 30:e13345. 10.1111/nmo.13345 29687532

[16] Zeng F Sun R He Z Chen Y Lei D Yin T. Altered functional connectivity of the amygdala and sex differences in functional dyspepsia. Clin Transl Gastroenterol. (2019) 10:e00046. 10.14309/ctg.0000000000000046 31136362 PMC6613861

[17] Liu X Li S-J Shaker R Silverman A Kern M Ward DB. Reduced functional connectivity between the hypothalamus and high-order cortical regions in adolescent patients with irritable bowel syndrome. J Pediatr Gastroenterol Nutr. (2017) 65:516–9. 10.1097/MPG.0000000000001611 29064927 PMC5657002

[18] Hong J-Y Kilpatrick LA Labus JS Gupta A Katibian D Ashe-McNalley C. Sex and disease-related alterations of anterior insula functional connectivity in chronic abdominal pain. J Neurosci. (2014) 34:14252–9. 10.1523/JNEUROSCI.1683-14.2014 25339739 PMC4205551

[19] Liu P Li G Zhang A Yang C Liu Z Sun N. Brain structural and functional alterations in MDD patient with gastrointestinal symptoms: a resting-state MRI study. J Affect Disord. (2020) 273:95–105. 10.1016/j.jad.2020.03.107 32421626

[20] Yan M Chen J Liu F Li H Huang R Tang Y. Disrupted regional homogeneity in major depressive disorder with gastrointestinal symptoms at rest. Front Psychiatry. (2021) 12:636820. 10.3389/fpsyt.2021.636820 34122171 PMC8187583

[21] Zou Q Zhu C Yang Y Zuo X Long X Cao Q. An improved approach to detection of amplitude of low-frequency fluctuation (ALFF) for resting-state fMRI: fractional ALFF. J Neurosci Methods. (2008) 172:137–41. 10.1016/j.jneumeth.2008.04.012 18501969 PMC3902859

[22] Yan C Zang Y DPARSF. A MATLAB toolbox for “pipeline” data analysis of resting-state fMRI. Front Syst Neurosci. (2010) 4:13. 10.3389/fnsys.2010.00013 20577591 PMC2889691

[23] Chang C-C Lin C-J. LIBSVM: a library for support vector machines. ACM Trans Intell Syst Technol. (2011) 2:1–27. 10.1145/1961189.1961199

[24] Pinto-Sanchez MI Ford AC Avila CA Verdu EF Collins SM Morgan D. Anxiety and depression increase in a stepwise manner in parallel with multiple FGIDs and symptom severity and frequency. Am J Gastroenterol. (2015) 110:1038–48. 10.1038/ajg.2015.128 25964226

[25] Akkasheh G Kashani-Poor Z Tajabadi-Ebrahimi M Jafari P Akbari H Taghizadeh M. Clinical and metabolic response to probiotic administration in patients with major depressive disorder: a randomized, double-blind, placebo-controlled trial. Nutrition. (2016) 32:315–20. 10.1016/j.nut.2015.09.003 26706022

[26] Bravo JA Forsythe P Chew MV Escaravage E Savignac HM Dinan TG. Ingestion of Lactobacillus strain regulates emotional behavior and central GABA receptor expression in a mouse via the vagus nerve. Proc Natl Acad Sci U S A. (2011) 108:16050–5. 10.1073/pnas.1102999108 21876150 PMC3179073

[27] Desbonnet L Garrett L Clarke G Kiely B Cryan JF Dinan TG. Effects of the probiotic Bifidobacterium infantis in the maternal separation model of depression. Neuroscience. (2010) 170:1179–88. 10.1016/j.neuroscience.2010.08.005 20696216

[28] Geng J Yan R Shi J Chen Y Mo Z Shao J. Altered regional homogeneity in patients with somatic depression: a resting-state fMRI study. J Affect Disord. (2019) 246:498–505. 10.1016/j.jad.2018.12.066 30599374

[29] Doricchi F Macci E Silvetti M Macaluso E. Neural correlates of the spatial and expectancy components of endogenous and stimulus-driven orienting of attention in the Posner task. Cereb Cortex. (2010) 20:1574–85. 10.1093/cercor/bhp215 19846472

[30] Japee S Holiday K Satyshur MD Mukai I Ungerleider LG. A role of right middle frontal gyrus in reorienting of attention: a case study. Front Syst Neurosci. (2015) 9:23. 10.3389/fnsys.2015.00023 25784862 PMC4347607

[31] Gibbs-Gallagher N Palsson OS Levy RL Meyer K Drossman DA Whitehead WE. Selective recall of gastrointestinal-sensation words: evidence for a cognitive-behavioral contribution to irritable bowel syndrome. Am J Gastroenterol. (2001) 96:1133–8. 10.1111/j.1572-0241.2001.03759.x 11318007

[32] Jones MP Dilley JB Drossman D Crowell MD. Brain-gut connections in functional GI disorders: anatomic and physiologic relationships. Neurogastroenterol Motil. (2006) 18:91–103. 10.1111/j.1365-2982.2005.00730.x 16420287

[33] Hamilton JP Farmer M Fogelman P Gotlib IH. Depressive rumination, the default-mode network, and the dark matter of clinical neuroscience. Biol Psychiatry. (2015) 78:224–30. 10.1016/j.biopsych.2015.02.020 25861700 PMC4524294

[34] Lemogne C le Bastard G Mayberg H Volle E Bergouignan L Lehéricy S. In search of the depressive self: extended medial prefrontal network during self-referential processing in major depression. Soc Cogn Affect Neurosci. (2009) 4:305–12. 10.1093/scan/nsp008 19307251 PMC2728628

[35] Lemogne C Delaveau P Freton M Guionnet S Fossati P. Medial prefrontal cortex and the self in major depression. J Affect Disord. (2012) 136:e1–e11. 10.1016/j.jad.2010.11.034 21185083

[36] Belleau EL Treadway MT Pizzagalli DA. The impact of stress and major depressive disorder on hippocampal and medial prefrontal cortex morphology. Biol Psychiatry. (2019) 85:443–53. 10.1016/j.biopsych.2018.09.031 30470559 PMC6380948

[37] Burkhouse KL Jacobs RH Peters AT Ajilore O Watkins ER Langenecker SA. Neural correlates of rumination in adolescents with remitted major depressive disorder and healthy controls. Cogn Affect Behav Neurosci. (2017) 17:394–405. 10.3758/s13415-016-0486-4 27921216 PMC5366093

[38] Price DD Craggs JG Zhou Q Verne GN Perlstein WM Robinson ME. Widespread hyperalgesia in irritable bowel syndrome is dynamically maintained by tonic visceral impulse input and placebo/nocebo factors: evidence from human psychophysics, animal models, and neuroimaging. Neuroimage. (2009) 47:995–1001. 10.1016/j.neuroimage.2009.04.028 19375508 PMC2844701

[39] Guo W Cui X Liu F Chen J Xie G Wu R. Increased anterior default-mode network homogeneity in first-episode, drug-naive major depressive disorder: a replication study. J Affect Disord. (2018) 225:767–72. 10.1016/j.jad.2017.08.089 28938513

[40] Guo W Liu F Zhang J Zhang Z Yu L Liu J. Abnormal default-mode network homogeneity in first-episode, drug-naive major depressive disorder. PLoS ONE. (2014) 9:e91102. 10.1371/journal.pone.0091102 24609111 PMC3946684

[41] Northoff G. Psychopathology and pathophysiology of the self in depression — neuropsychiatric hypothesis. J Affect Disord. (2007) 104:1–14. 10.1016/j.jad.2007.02.012 17379318

[42] Hasler G Northoff G. Discovering imaging endophenotypes for major depression. Mol Psychiatry. (2011) 16:604–19. 10.1038/mp.2011.23 21602829

[43] Araujo HF Kaplan J Damasio A. Cortical midline structures and autobiographical-self processes: an activation-likelihood estimation meta-analysis. Front Hum Neurosci. (2013) 7:548. 10.3389/fnhum.2013.00548 24027520 PMC3762365

[44] Phan KL Wager T Taylor SF Liberzon I. Functional neuroanatomy of emotion: a meta-analysis of emotion activation studies in PET and fMRI. Neuroimage. (2002) 16:331–48. 10.1006/nimg.2002.1087 12030820

[45] Zhou H-X Chen X Shen Y-Q Li L Chen N-X Zhu Z-C. Rumination and the default mode network: meta-analysis of brain imaging studies and implications for depression. Neuroimage. (2020) 206:116287. 10.1016/j.neuroimage.2019.116287 31655111

[46] Phillips ML Drevets WC Rauch SL Lane R. Neurobiology of emotion perception II: implications for major psychiatric disorders. Biol Psychiatry. (2003) 54:515–28. 10.1016/S0006-3223(03)00171-9 12946880

[47] Levinthal DJ Strick PL. Multiple areas of the cerebral cortex influence the stomach. Proc Natl Acad Sci U S A. (2020) 117:13078–83. 10.1073/pnas.2002737117 32434910 PMC7293610

[48] Zhao D-Q Xue H Sun H-J. Nervous mechanisms of restraint water-immersion stress-induced gastric mucosal lesion. World J Gastroenterol. (2020) 26:2533–49. 10.3748/wjg.v26.i20.2533 32523309 PMC7265141

[49] Wang H Guo W Liu F Chen J Wu R Zhang Z. Clinical significance of increased cerebellar default-mode network connectivity in resting-state patients with drug-naive somatization disorder. Medicine. (2016) 95:e4043. 10.1097/MD.0000000000004043 27428190 PMC4956784

[50] Su Q Yao D Jiang M Liu F Jiang J Xu C. Dissociation of regional activity in default mode network in medication-naive, first-episode somatization disorder. PLoS ONE. (2014) 9:e99273. 10.1371/journal.pone.0099273 24983962 PMC4077566

[51] Seifert F Bschorer K De Col R Filitz J Peltz E Koppert W. Medial prefrontal cortex activity is predictive for hyperalgesia and pharmacological antihyperalgesia. J Neurosci. (2009) 29:6167–75. 10.1523/JNEUROSCI.4654-08.2009 19439594 PMC6665503

[52] Qi R Liu C Ke J Xu Q Zhong J Wang F. Intrinsic brain abnormalities in irritable bowel syndrome and effect of anxiety and depression. Brain Imaging Behav. (2016) 10:1127–34. 10.1007/s11682-015-9478-1 26556814

[53] Coutinho SV Plotsky PM Sablad M Miller JC Zhou H Bayati AI. Neonatal maternal separation alters stress-induced responses to viscerosomatic nociceptive stimuli in rat. Am J Physiol Gastrointest Liver Physiol. (2002) 282:G307–316. 10.1152/ajpgi.00240.2001 11804852

[54] Bradesi S Schwetz I Ennes HS Lamy CMR Ohning G Fanselow M. Repeated exposure to water avoidance stress in rats: a new model for sustained visceral hyperalgesia. Am J Physiol Gastrointest Liver Physiol. (2005) 289:G42–53. 10.1152/ajpgi.00500.2004 15746211

[55] Wu JCY. Community-based study on psychological comorbidity in functional gastrointestinal disorder. J Gastroenterol Hepatol. (2011) 26:23–6. 10.1111/j.1440-1746.2011.06642.x 21443703

[56] Bhattarai Y Muniz Pedrogo DA Kashyap PC. Irritable bowel syndrome: a gut microbiota-related disorder? Am J Physiol Gastrointest Liver Physiol. (2017) 312:G52–62. 10.1152/ajpgi.00338.2016 27881403 PMC5283907

[57] Grieve SM Korgaonkar MS Koslow SH Gordon E Williams LM. Widespread reductions in gray matter volume in depression. Neuroimage Clin. (2013) 3:332–9. 10.1016/j.nicl.2013.08.016 24273717 PMC3814952

[58] Maller JJ Thomson RHS Rosenfeld JV Anderson R Daskalakis ZJ Fitzgerald PB. Occipital bending in depression. Brain. (2014) 137:1830–7. 10.1093/brain/awu072 24740986

[59] Yao X Yin Z Liu F Wei S Zhou Y Jiang X. Shared and distinct regional homogeneity changes in bipolar and unipolar depression. Neurosci Lett. (2018) 673:28–32. 10.1016/j.neulet.2018.02.033 29466722

[60] Guo W Liu F Xue Z Xu X Wu R Ma C. Alterations of the amplitude of low-frequency fluctuations in treatment-resistant and treatment-response depression: A resting-state fMRI study. Prog Neuropsychopharmacol Biol Psychiatry. (2012) 37:153–60. 10.1016/j.pnpbp.2012.01.011 22306865

[61] Meng C Brandl F Tahmasian M Shao J Manoliu A Scherr M. Aberrant topology of striatum's connectivity is associated with the number of episodes in depression. Brain. (2014) 137:598–609. 10.1093/brain/awt290 24163276

[62] Epperson CN Gueorguieva R Czarkowski KA Stiklus S Sellers E Krystal JH. Preliminary evidence of reduced occipital GABA concentrations in puerperal women: a 1H-MRS study. Psychopharmacology. (2006) 186:425–33. 10.1007/s00213-006-0313-7 16724188

[63] Sanacora G Mason GF Rothman DL Krystal JH. Increased occipital cortex GABA concentrations in depressed patients after therapy with selective serotonin reuptake inhibitors. Am J Psychiatry. (2002) 159:663–5. 10.1176/appi.ajp.159.4.663 11925309

[64] Alders GL Davis AD MacQueen G Strother SC Hassel S Zamyadi M. Reduced accuracy accompanied by reduced neural activity during the performance of an emotional conflict task by unmedicated patients with major depression: a CAN-BIND fMRI study. J Affect Disord. (2019) 257:765–73. 10.1016/j.jad.2019.07.037 31400735

[65] Quevedo K Harms M Sauder M Scott H Mohamed S Thomas KM. The neurobiology of self face recognition among depressed adolescents. J Affect Disord. (2018) 229:22–31. 10.1016/j.jad.2017.12.023 29304386 PMC5898821

[66] Chechko N Kellermann T Zvyagintsev M Augustin M Schneider F Habel U. Brain circuitries involved in semantic interference by demands of emotional and non-emotional distractors. PLoS ONE. (2012) 7:e38155. 10.1371/journal.pone.0038155 22666470 PMC3362560

[67] Weiner KS Zilles K. The anatomical and functional specialization of the fusiform gyrus. Neuropsychologia. (2016) 83:48–62. 10.1016/j.neuropsychologia.2015.06.033 26119921 PMC4714959

[68] Liu P Li Y Zhang A-X Sun N Li G-Z Chen X. Brain structural alterations in MDD patients with gastrointestinal symptoms: evidence from the REST-meta-MDD project. Prog Neuropsychopharmacol Biol Psychiatry. (2021) 111:110386. 10.1016/j.pnpbp.2021.110386 34119573

[69] Guo W Liu F Xue Z Yu Y Ma C Tan C. Abnormal neural activities in first-episode, treatment-naïve, short-illness-duration, and treatment-response patients with major depressive disorder: a resting-state fMRI study. J Affect Disord. (2011) 135:326–31. 10.1016/j.jad.2011.06.048 21782246

[70] Maggioni E Delvecchio G Grottaroli M Garzitto M Piccin S Bonivento C. Common and different neural markers in major depression and anxiety disorders: a pilot structural magnetic resonance imaging study. Psychiatry Res Neuroimaging. (2019) 290:42–50. 10.1016/j.pscychresns.2019.06.006 31279954

[71] Wang Y Zhong S Jia Y Zhou Z Wang B Pan J. Interhemispheric resting state functional connectivity abnormalities in unipolar depression and bipolar depression. Bipolar Disord. (2015) 17:486–95. 10.1111/bdi.12315 26241359

[72] Yrondi A Nemmi F Billoux S Giron A Sporer M Taib S. Grey matter changes in treatment-resistant depression during electroconvulsive therapy. J Affect Disord. (2019) 258:42–9. 10.1016/j.jad.2019.07.075 31382103

[73] Gong J Wang J Qiu S Chen P Luo Z Wang J. Common and distinct patterns of intrinsic brain activity alterations in major depression and bipolar disorder: voxel-based meta-analysis. Transl Psychiatry. (2020) 10:353. 10.1038/s41398-020-01036-5 33077728 PMC7573621

