# Supplementary material for: Shared and Distinct Fractional Amplitude of Low-Frequency Fluctuation Patterns in Major Depressive Disorders With and Without Gastrointestinal Symptoms
Source: Front Psychiatry. 2021 Dec 2;12:744898. doi: 10.3389/fpsyt.2021.744898 (PMC8674438; doi:10.3389/fpsyt.2021.744898)
Supplement: Supplementary file 1 [file Data_Sheet_1.pdf]

**Supplementary Online Content**

**Figure S1.** Regional fALFF differences between GI-MDD patients and nGI-MDD patients, with age, sex ratio, years of education, the mean FD and the HRSD-17 score were set as covariates.

**Table S1.** Significant fALFF differences between GI-MDD and nGI-MDD

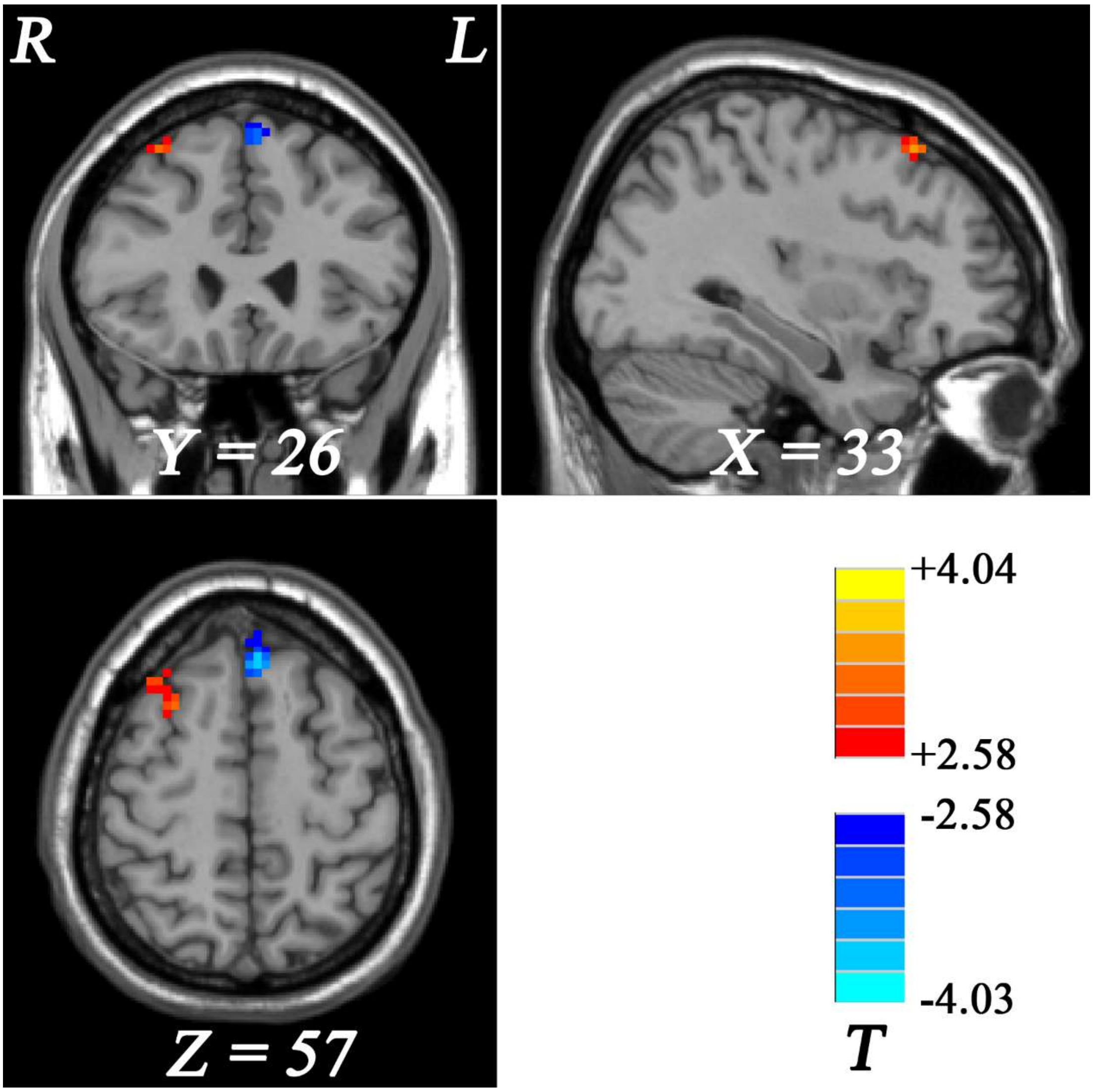


**Figure S1.** Regional fALFF differences between GI-MDD patients and nGI-MDD patients, with age, sex ratio, years of education, the mean FD and the HRSD-17 score were set as covariates. Red and blue colors denote increased and decreased fALFF, respectively. fALFF: fractional amplitude of low-frequency fluctuation, FD: framewise displacement, GI-MDD: major depressive disorder with gastrointestinal symptoms, nGI-MDD: major depressive disorder without gastrointestinal symptoms.

**Table S1.** Significant fALFF differences between GI-MDD and nGI-MDD

| Cluster location | Peak (MNI) | | | Number of voxels | *T* value |
|---|---|---|---|---|---|
| | x | y | z | | |
| *S1 vs S0 (Figure S1)* | | | | | |
| Right Superior Frontal Gyrus/Middle Frontal Gyrus | 33 | 24 | 54 | 25 | 3.3497 |
| Left Superior MPFC | -3 | 30 | 57 | 40 | -3.6995 |

MNI = Montreal Neurological Institute; MPFC = Medial Prefrontal Cortex.

S1: MDD patients with gastrointestinal (GI) symptoms, S0: MDD patients without GI symptoms.
